# Forensic age estimation from human blood using age-related microRNAs and circular RNAs markers

**DOI:** 10.3389/fgene.2022.1031806

**Published:** 2022-11-22

**Authors:** Junyan Wang, Haixia Zhang, Chunyan Wang, Lihong Fu, Qian Wang, Shujin Li, Bin Cong

**Affiliations:** ^1^ College of Forensic Medicine, Hebei Medical University, Hebei Key Laboratory of Forensic Medicine, Collaborative Innovation Center of Forensic Medical Molecular Identification, Research Unit of Digestive Tract Microecosystem Pharmacology and Toxicology, Chinese Academy of Medical Sciences, Shijiazhuang, Hebei, China; ^2^ Physical Examination Center of Shijiazhuang First Hospital, Shijiazhuang, Hebei, China

**Keywords:** forensic genetics, age estimation, non-coding RNAs, bioinformatics, machine learning

## Abstract

Aging is a complicated process characterized by progressive and extensive changes in physiological homeostasis at the organismal, tissue, and cellular levels. In modern society, age estimation is essential in a large variety of legal rights and duties. Accumulating evidence suggests roles for microRNAs (miRNAs) and circular RNAs (circRNAs) in regulating numerous processes during aging. Here, we performed circRNA sequencing in two age groups and analyzed microarray data of 171 healthy subjects (17–104 years old) downloaded from Gene Expression Omnibus (GEO) and ArrayExpress databases with integrated bioinformatics methods. A total of 1,403 circular RNAs were differentially expressed between young and old groups, and 141 circular RNAs were expressed exclusively in elderly samples while 10 circular RNAs were expressed only in young subjects. Based on their expression pattern in these two groups, the circular RNAs were categorized into three classes: age-related expression between young and old, age-limited expression-young only, and age-limited expression-old only. Top five expressed circular RNAs among three classes and a total of 18 differentially expressed microRNAs screened from online databases were selected to validate using RT-qPCR tests. An independent set of 200 blood samples (20–80 years old) was used to develop age prediction models based on 15 age-related noncoding RNAs (11 microRNAs and 4 circular RNAs). Different machine learning algorithms for age prediction were applied, including regression tree, bagging, support vector regression (SVR), random forest regression (RFR), and XGBoost. Among them, random forest regression model performed best in both training set (mean absolute error = 3.68 years, r = 0.96) and testing set (MAE = 6.840 years, r = 0.77). Models using one single type of predictors, circular RNAs-only or microRNAs-only, result in bigger errors. Smaller prediction errors were shown in males than females when constructing models according to different-sex separately. Putative microRNA targets (430 genes) were enriched in the cellular senescence pathway and cell homeostasis and cell differentiation regulation, indirectly indicating that the microRNAs screened in our study were correlated with development and aging. This study demonstrates that the noncoding RNA aging clock has potential in predicting chronological age and will be an available biological marker in routine forensic investigation to predict the age of biological samples.

## 1 Introduction

Forensic age estimation in the identification of unknown deceased and unknown donors of a trace can provide valuable clues for the police and investigators to narrow down the investigation scope and clear the cognizance of potential criminal suspects. Many conventional morphological methods such as measuring bones (Scendoni et al., 2020) or teeth (Koh et al., 2017) have been expanded to various new strategies based on the use of age-related molecular changes such as analyzing the amino acid racemization of teeth (Matteussi et al., 2022) and other tissue specimens such as skin and cartilage tissues (Tiplamaz et al., 2018). However, these methods have limitations in cases where a skeleton is not present. It is desirable to use molecular methods that allow retrieving a donor’s age information from various human biological samples including body fluids such as blood, which are amongst the most important biological evidence recovered from crime scenes (Zubakov et al., 2016).

To date, numerous studies have investigated the ability of molecular indicators in blood to predict chronological age in forensic examinations, including telomere shortening (Vaiserman and Krasnienkov, 2020), mitochondrial DNA deletion(Zapico and Ubelaker, 2016), signal-joint T-cell receptor excision circle (sjTRECs) (Yamanoi et al., 2018), and others. The first DNA methylation (DNAm) epigenetic aging clock was developed by Hannum in 2013 (Hannum et al., 2013). For a long time, DNAm became a “black spot” for forensic scientists (Naue, et al., 2017). DNAm, which has emerged as a most promising method for predicting age in forensics with an uncertainty mean absolute deviation of about 3–5 years in the predicted age, provides high accuracy but has several limitations such as requiring relatively large amounts of DNA and complicated bioinformatics analysis (Park et al., 2016). The quantity and quality of DNA in postmortem cases and traces in crime scene investigations are often restricted (Koop et al., 2021). Age estimation in these circumstances is confined, which pushes researchers to search for novel tools to aid in forensic practice as a supplemental method. Recently, some potential candidate biomarkers for age estimation have come into play. Accumulating evidence suggests roles for microRNAs (miRNAs) and circular RNAs (circRNAs) in regulating a large variety of processes during aging. These non-coding RNAs (ncRNAs) generally act as post-transcriptional regulators of gene expression. Specifically, miRNAs either suppress translation or degrade the targeted mRNA, or both, by binding to miRNA-recognition elements (MREs) in target transcripts (Victoria et al., 2017). While circRNAs regulate gene expression, *via* interaction with miRNAs and RNA binding proteins as molecular sponges (Hansen et al., 2013; Memczak et al., 2013).

MiRNAs are a class of small ncRNAs, short and single-stranded, typically 18–22 nt in length with post-transcriptionally regulatory functions. Lin-4 was the first miRNA discovered by Lee et al., in 1993 with the finding of its functional ability to regulate protein production (Lee et al., 1993). Whereas the first forensic application of miRNAs was in 2009. Hanson et al. proposed miRNAs as potential biomarkers for forensic body fluid identification (Hanson et al., 2009). Numerous experimental data demonstrated that miRNAs involve in the modulation of physiological and pathophysiological aging, for instance, Zhang et al. (Zhang et al., 2015) identified significantly down-regulated age-dependent miRNAs in serum miRNA profiles from adults (40–70 years old), such as miR-29b, miR-106b; and up-regulated miRNAs, such as miR-92a, miR-222, and miR-375. Therefore, some forensic scientists proposed the potential forensic application of miRNAs in estimating the age of a donor of biological samples. Fang et al. (Fang et al., 2020) established age prediction models for bloodstains based on six age-related miRNAs (miR-98-3p, miR-324-3p, miR-32-3p, miR-330-5p, miR-374c-5p and miR-342-3p) using seven machine learning models. Results showed that the mean absolute error (MAE) ranged from 6.56 to 9.262 years. Fang’s study elucidated the possibility of performing forensic age prediction using miRNAs. CircRNAs are another class of ncRNAs newly discovered in recent years, originating through back-splicing events from linear primary transcripts and presenting as a special covalent loop without a 5′ cap or 3′ tail (Cai et al., 2019). They are resistant to RNase R activity since they are covalently closed, giving rise to their stability than linear RNA. CircRNAs are widely expressed in eukaryotes and can function as miRNA sponges. Recent reports showed that there is an accumulated trend for circRNA expression in various species (Gruner et al., 2016; Du et al., 2017; Cortes-Lopez et al., 2018; TONG ZHOU et al., 2018) and could play a role in aging, such as neural aging (Zhang et al., 2022), muscle aging (Cai et al., 2019), skin aging (Wang et al., 2021), and age-related diseases like Alzheimer’s disease (Mo et al., 2020; D'Anca et al., 2022; Liu et al., 2022). CircRNAs have emerged as promising candidate biomarkers in aging due to their expression patterns and stability (Cai et al., 2019; Pan et al., 2020). Both miRNAs and circRNAs have shown satisfactory stability and are highly correlated with age (Glynn, 2020; Zhao et al., 2021), we suggest they can be considered as biological age predictors. In our previous work (Wang et al., 2022), we developed several age predictive models to investigate the potential of circRNAs in predicting chronological age in human blood. Results showed a relatively big error with MAE values of 8.77–12.19 years, which cannot meet the demands of forensic practice.

The purpose of our study was to identify circRNAs with better correlations with age, building age predictive models with higher accuracy. We considered combinations of different age-related predictors to reduce predictive errors, thus we searched for age-related miRNAs from public databases. Here, we identified age-related circRNAs by comparing circRNA expression profiles of elder subjects (50–62 years old) to young subjects (20–29 years old) using circRNA next-generation sequencing (NGS). Age-related miRNAs were screened using integrated bioinformatics methods. miRNA microarray data of 171 samples (17–104 years old) was downloaded from Gene Expression Omnibus (GEO) and Arrayexpress databases. Age-associated ncRNAs were validated utilizing RT-qPCR assays. Regression models were developed and evaluated using multiple machine learning algorithms based on 200 samples (20–80 years old).

## 2 Materials and methods

### 2.1 Participants and RNA extraction

The participants of this study were classified into three independent sets: a screening set, a validation set and a modeling set. The screening set consisted of four young subjects (20–29 years) and four elderly subjects (50–62 years) from circRNA-seq and 171 samples of miRNA microarray from public datasets. The validation set consisted of 40 samples aged between 19–73 years old for the evaluation of age-related ncRNAs using RT-qPCR. The modeling set was used for predictive models’ construction, including an independent cohort of blood samples collected from 200 volunteers of 20–80 years of age (102 females and 98 males). Blood samples (10 ml) were drawn by venipuncture from 248 healthy unrelated subjects with informed consent. The age distribution and sex distribution of 248 samples were displayed in Supplementary Figure S1. Our study was approved by the Medical Ethics Committee of Hebei Medical University (No. 20190013).

Three hundred microliters of the whole blood samples were used for RNA extraction using nine hundred microliters TRIzol reagent (Thermo Scientific, United States) according to the manufacturer’s instructions. NanoDrop ND-1000 (NanoDrop Technologies) was used for RNA quantification. RNA integrity was assessed on the Bioanalyzer 2100 system (Agilent Technologies, CA, United States).

### 2.2 Circular RNAs next-generation sequencing

A total amount of 5 μg RNA per sample was used as input material for the RNA sample preparations. Libraries were prepared and generated using NEBNext^®^ UltraTM Directional RNA Library Prep Kit for Illumina^®^ (NEB, United States) following the manufacturer’s recommendations. Barcoded libraries were sequenced at Novogene Co., LTD. (Beijing, China) on an Illumina HiSeq 4,000 platform and 150 bp paired-end reads were generated. Raw data of fastq format were firstly processed to obtain clean reads. Clean data (clean reads) were obtained by removing reads containing adapter, ploy-N and low-quality reads from raw data. All the downstream analyses were based on clean data with high quality. Subsequently, paired-end clean reads were aligned to the reference genome (hg38) using Bowtie (Langmead et al., 2009). Find_circ (Memczak et al., 2013) and CIRI2 (Gao et al., 2015) were used to detect and identify circRNAs. To account for variability due to differences in library size, counts attributes to individual circRNAs were normalized to total read count to obtain circRNA TPM values. Raw FASTQ files from the RNA-seq data were deposited at the NCBI Sequence Read Archive (BioProject: PRJNA682456).

### 2.3 Microarray data

The inclusion criteria of public datasets were 1) studies conducted in human blood samples; 2) researches using miRNA microarray technology; 3) a sample size of at least 30 healthy subjects of different ages; 4) publication before 2020 and restricted to reporting in the English language. There are three public datasets were selected from the ArrayExpress and the GEO databases. The miRNA microarray datasets were preprocessed through several steps: normalization, log2 transformation, batch effect removement, filtering and ID annotation of probes. The ordinary data of two datasets were downloaded from the ArrayExpress database. The dataset E-MTAB-1231 used Affymetrix GeneChip miRNA 2.0 Array and E-MTAB-3303 was performed on Illumina Human microRNA V2 BeadChip. R packages “*oligo*” and “*limma*” were applied to calibrate, normalize, remove batch effects and log2 transform the raw data, respectively. The series matrix files and platform information of one microarray dataset with accession GSE89042 were downloaded from the NCBI GEO database. We analyzed them with the R package “*Biobase*” for normalization and log2 transformation. The basic information of three public datasets was listed in Supplementary Table S1.

### 2.4 Reverse transcription and quantification

For age-related circRNAs, total RNAs were reverse transcribed into cDNAs using the PrimerScript Reverse Transcription Kit with gDNA Eraser (Takara Bio Inc.) and cDNAs were quantified using the QuantiNova™ SYBR Green PCR kit. For age-related miRNAs, total RNAs were treated with DNase Ⅰ to remove genomic DNA using Recombinant DNaseⅠ Kit (Takara Bio Inc.). cDNA synthesis and quantitation were performed utilizing Mir-X™ miRNA First-Strand Synthesis and TB Green qRT-PCR Kit (Takara Bio Inc.). Both age-related circRNAs and miRNAs were quantified on an ABI 7500 real-time PCR system according to the manufacturer’s protocol. The relative expression level of each ncRNA was calculated using the normalized threshold cycle number (ΔC_t_), in which ΔC_t_ = [C_t_ (ncRNA)—[C_t_ (reference gene)]. Sequences of primers used in our study are available in Supplementary Table S2.

### 2.5 Statistical analyses

#### 2.5.1 Screening of age-related circular RNAs

The relationship between circRNA expressivity and age was used to classify the circRNAs into three classes: age-limited expression-young only, age-limited expression-old only, and age-related expression. To select the differentially expressed circRNAs between young and old, the “*limma*” package in the Bioconductor package (http://www.bioconductor.org/) was used to perform the selection of age-related differentially expressed circRNAs. CircRNAs satisfied the following criteria were considered as age-related candidates: absolute-value of log (foldchange) > 2 and *p*-value < 0.001.

#### 2.5.2 Selection of age-related microRNAs

The Spearman correlation method was used to screen age-related miRNAs for datasets GSE89042 and E-MTAB-3303, in which the response variable (i.e. age) is a continual variable. MiRNAs with an absolute Spearman’s correlation coefficient value >0.2 and an adjusted *p*-value < 0.05 were considered as age-related candidate biomarkers for subsequent analyses. Dataset E-MTAB-1231 consisted of 50 samples which can be naturally classified into the young group (<40 years old) and the old group (70–104 years old). Thus, we used the “*limma*” package in the Bioconductor package to perform differential expression analysis to select age-related miRNAs. MiRNA candidates matched the criteria as follows: absolute-value of log(foldchange) > 2 and adjusted *p*-value < 0.05. The lists of age-associated miRNAs screened from three datasets were saved as TXT files.

#### 2.5.3 Age-related microRNAs and circular RNAs used for experimental validation

An independent set of samples was used to experimentally evaluate ncRNAs screened from circRNA-seq and public databases. The top five differentially expressed circRNAs among three classes that showed age-limited or age-related expression, and miRNAs differentially expressed in at least two datasets as age-related ncRNAs were chosen for validation using the RT-qPCR strategy. ncRNAs with an absolute Spearman’s correlation coefficient value >0.2 were selected for further work. Statistical analyses were performed using IBM SPSS Statistics V.21, Graph Pad Prism (Version 8.0, Inc., CA, United States) and R software (4.1.2).

### 2.6 MicroRNAs targets prediction and functional enrichment analysis

Three online databases miRTarbase (http://mirtarbase.mbc.nctu.edu.tw/), miRWalk (http://mirwalk.umm.uni-heidelberg.de/), TargetScan (http://www.targetscan.org/vert_72/) were used to search miRNA target genes, including predicted genes and validated genes. The putative miRNA targets from three databases were intersected to obtain the list of target genes for further functional and pathway analysis.

Gene Ontology (GO) and Kyoto Encyclopedia of Genes and Genomes (KEGG) pathway analyses were conducted for miRNA targets using the “*clusterProfiler”* package in R software. The cut-off criterion was set as an adjusted *p*-value of <0.05. Both GO and KEGG enrichment analyses outcomes were visualized using “*GOplot*” in the R package.

### 2.7 Construction of age predictive models

Age-related ncRNAs experimentally validated were used for age predictive model construction in additional 200 blood samples. All samples were randomly divided into two sets: a training set (80% of all subjects) to construct the age-predictive model and a testing set (the remaining 20%) to evaluate the model’s prediction performance. Several machine learning algorithms were applied to fit models, including regression tree, bagging, random forest regression (RFR), support vector regression (SVR) and XGBoost. R project for statistical computing software version 4.1.2 was employed using *rpart, ipred, randomForest, e1071, xgboost* packages, respectively. To determine the performance of different models, the root mean square error (RMSE) and mean absolute error (MAE) from the chronological age were calculated for the testing set.

### 2.8 Sex effect analysis

For the analyses of the relationship between age-related ncRNAs expression and sex effect, statistical comparisons between males and females were performed using nonparametric t-tests (Mann-Whitney test) in Graph Pad Prism (Version 8.0, Inc., CA, United States). Additionally, the female and male samples were used for modeling separately using abovementioned machine learning algorithms to investigate whether sex effect have an impact on the accuracy of age prediction models in our study.

## 3 Results

**FIGURE 1:**
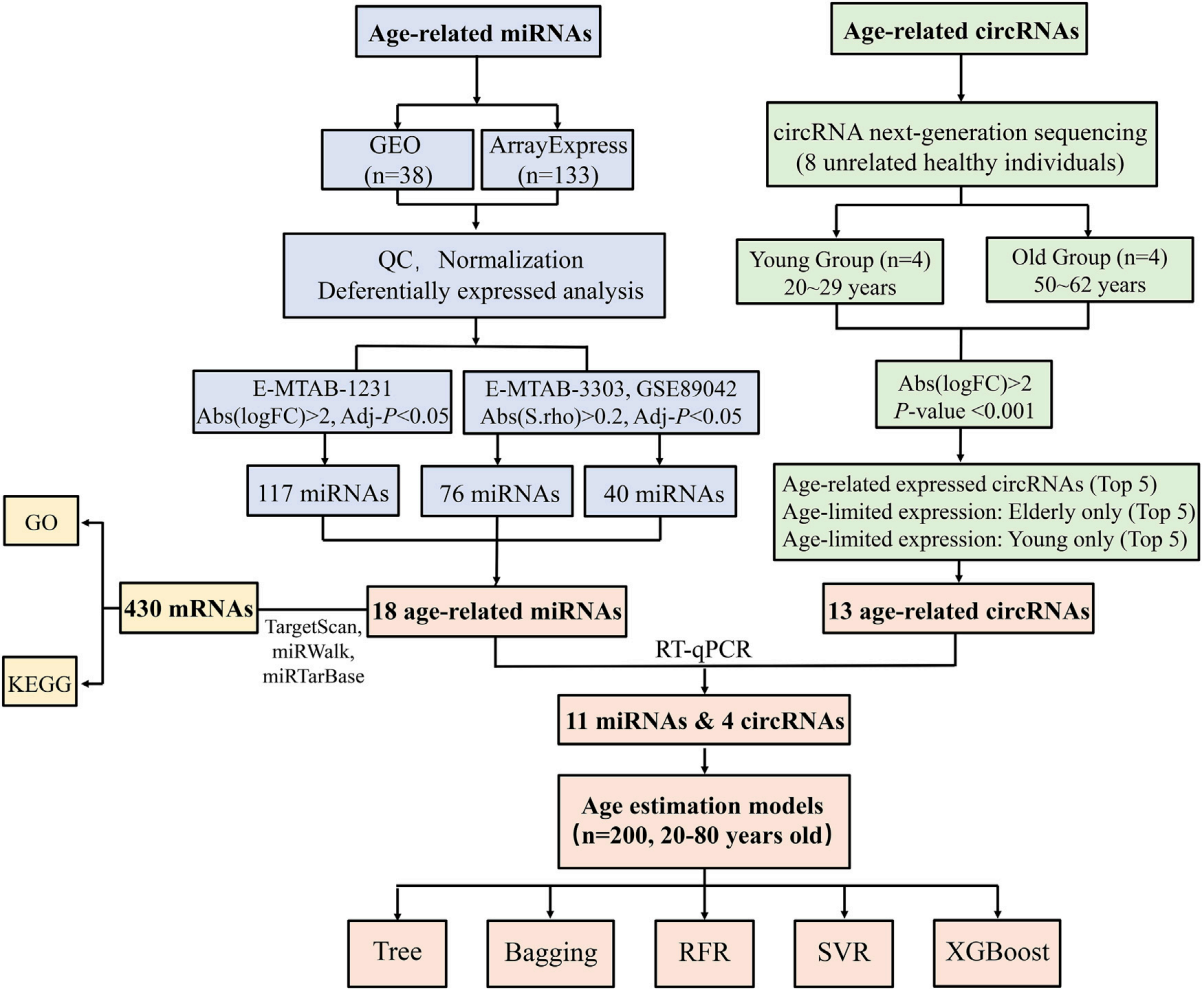
Flow chart of this study.

Figure 1 depicts the workflow of our study. In this study, we conducted circRNA-seq and analyzed several miRNA microarray datasets with integrated bioinformatic methods. 15 age-related ncRNAs (11 miRNAs and 4 circRNAs) were identified to construct age estimation models. These differentially expressed ncRNA biomarkers may play a role in forensic age prediction.

### 3.1 Circular RNAs expression profiles and microRNAs microarray preprocessing

Results output an average of 93.8 million total reads and an average of 78.3 million mapped reads per sample. A total of 43,325 circRNAs were identified in young and old individuals. A vast majority of circRNA (>85%) were generated from annotated protein-coding genes. Comparing young with old, circRNAs were classified into three classes: 1) age-related expressed circRNAs: a total of 1,403 circRNAs were differentially expressed. 2) age-limited expression-old only (141 circRNAs, 10%). 3) age-limited expression-young only (10 circRNAs, 0.7%). Box plots of quality assessment were shown in Figures S2.

### 3.2 Age-related non-coding RNAs selection and experimental validation

Of the 1,403 circRNAs that were differentially expressed in young and old samples, 921 exhibited increasing expression from young to old, and 482 showed a decreasing trend. Among them, 27 circRNAs with a *p*-value < 0.001. We finally selected 13 circRNAs for RT-qPCR validation: the top five expressed exclusively in the young subjects; the top five expressed exclusively in the old subjects; and the top five expressed in both groups but showed the most discrepant expression (two circRNAs were overlapped). However, 4 circRNAs (circCAMLG, circNCOA5, circCNTRL and circBMPR2) were excluded because they failed in the primer design and one (circNFATC3) was eliminated due to barely detectable (with Ct values higher than 37). Therefore, there were a total of eight age-related circRNAs used for further analyses. We chose miRNAs differentially expressed in at least two datasets as age-related miRNAs. The dataset E-MTAB-1231 contained 117 age-related miRNAs all upregulated expressed with age. The GSE89042 dataset screened 40 differentially expressed miRNAs, including 30 upregulated miRNAs and 10 downregulated miRNAs from young to old. A total of 55 miRNAs were significantly differentially expressed in dataset E-MTAB-3303, including 40 upregulated and 15 downregulated miRNAs (Supplementary Table S1). A set of 26 overlapping age-related miRNAs were identified, however, 8 miRNAs were excluded due to their inconsistent expression trend between different datasets. Their expression patterns and locations on human chromosomes were presented in Supplementary Table S3. Finally, 18 age-related miRNAs were selected for further experimental validation.

An independent cohort of samples was used to experimentally evaluate ncRNAs (8 circRNAs and 18 miRNAs) screened from circRNA-seq and public databases. Finally, 4 circRNAs and 11 miRNAs with an absolute Spearman’s correlation coefficient >0.2 were chosen for modelling: hsa_circ_0104,147, hsa_circ_0005400, circMYH11, circPPP2R5A, miR-107, miR-339-5p, miR-940, miR-27a-3p, miR-1281, miR-1228, miR-330-3p, miR-491-5p, miR-500a-3p, miR-222 and miR-744. Most of these ncRNAs were upregulated with age and the RT-qPCR validation was consistent with NGS or microarray results, except for circRNA hsa_circ_0005400, which was downregulated in NGS but significantly upregulated in RT-qPCR.

### 3.3 Identification of target genes and functional analysis

**FIGURE 2:**
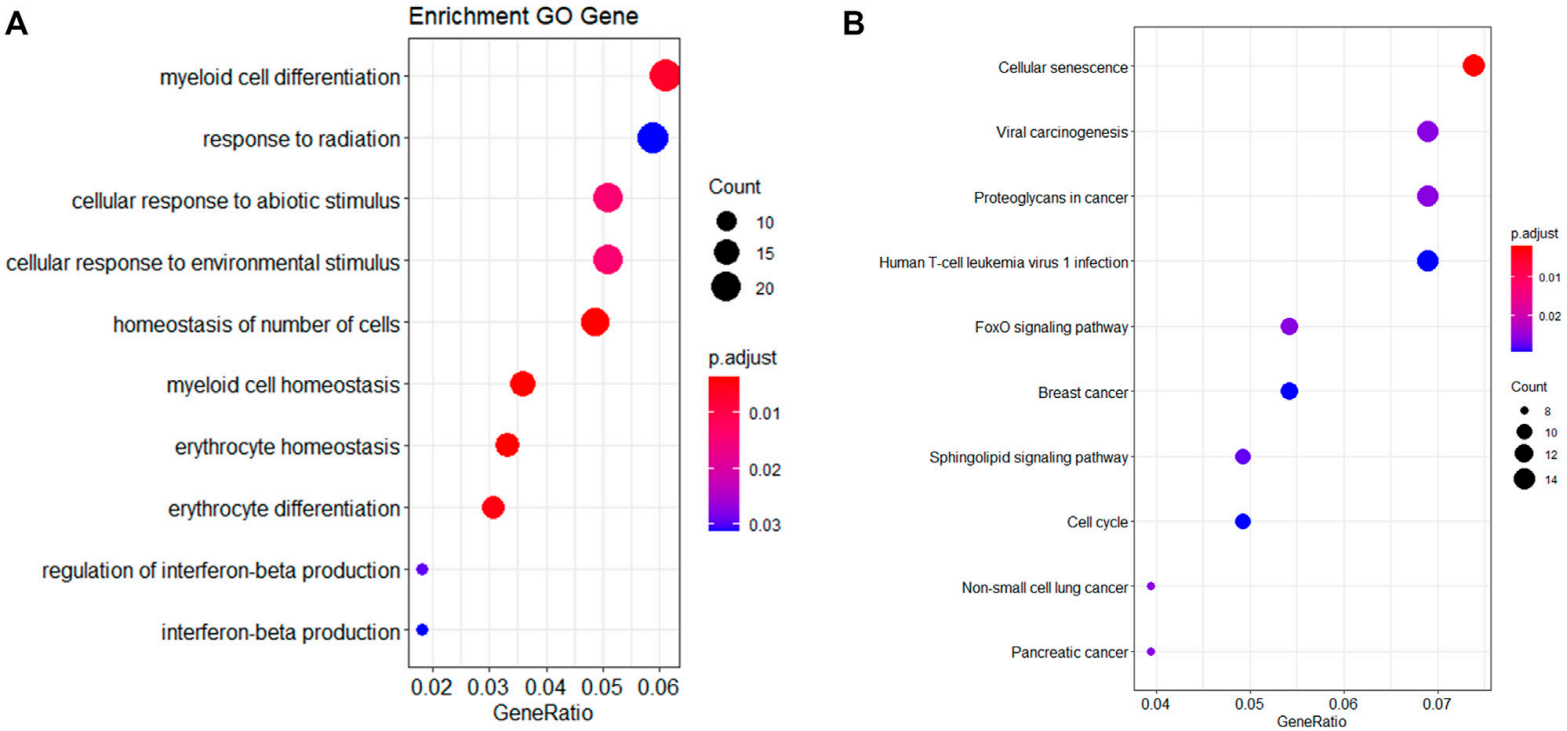
GO annotation **(A)** and KEGG pathway enrichment **(B)** of miRNA target genes.

Each age-related miRNA was predicted target genes according to the intersection of three databases (miRTarbase, miRWalk, and TargetScan). A list of 430 target genes was identified, which was input into R for GO and KEGG analyses. Analysis revealed the top 10 most enriched GO terms, including homeostasis of the number of cells, erythrocyte homeostasis, myeloid cell homeostasis, erythrocyte differentiation, myeloid cell differentiation, cellular response to abiotic stimulus, cell response to environmental stimulus, regulation of interferon-beta production, response to radiation and interferon-beta production, as represented in Figure 2A and Supplementary Table S4. A KEGG pathway analysis showed target genes mainly enriched in cellular senescence, and FOXO signaling pathway, as displayed in Figure 2B and Supplementary Table S5.

### 3.4 Development and evaluation of age prediction models using several algorithms

**FIGURE 3:**
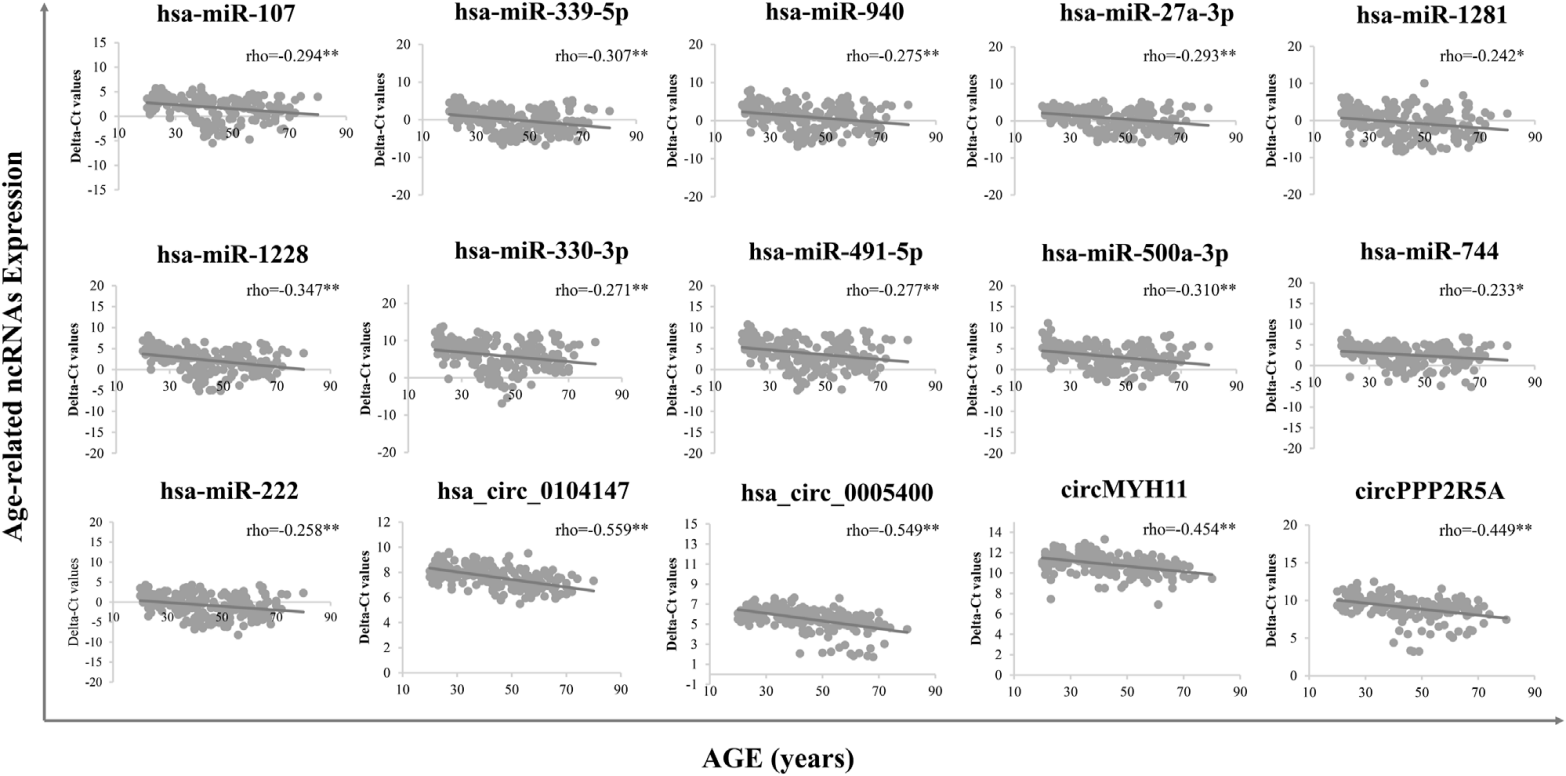
Final age-dependent markers in 200 blood samples from real-time PCR. Delta-Ct values were used to represent the relative expression levels of age-related ncRNAs.

**FIGURE 4:**
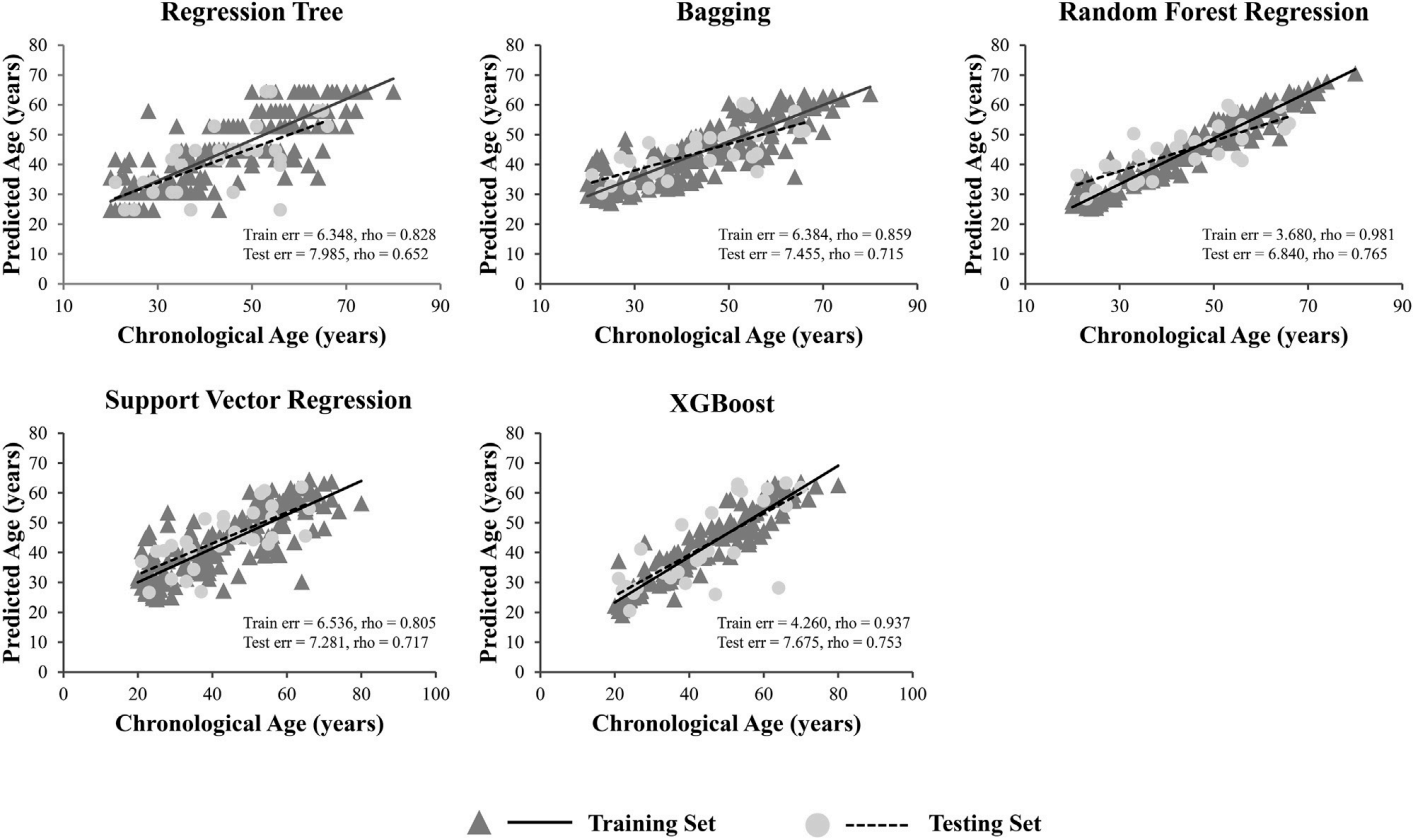
Predicted *versus* chronological ages using regression tree, bagging, RFR, SVR and XGBoost.

A total of 15 age-related ncRNAs (4 circRNAs and 11 miRNAs) quantified in an independent set of 200 blood samples aged from 20 to 80 years old was used to develop the age prediction models. CircRNAs hsa_circ_0104147 and hsa_circ_0005400 in 200 samples showed a significant correlation with age (Spearman’s rho >0.5), while in general, miRNAs showed a relatively weak positive correlation with age (Spearman’s rho: 0.2–0.4) compared to circRNAs (Figure 3). The 200 samples were randomly divided into a training set (80%) and a testing set (20%). Several machine learning methods were introduced, including regression tree, bagging, RFR, SVR, and XGBoost. The MAE values for the training set ranged from 3 to 6 years, while 6–7 years for the testing set (Table 1). Among them, RFR performed best, followed by SVR and bagging. The RFR model trained with age information and ncRNAs expression pattern explained 92.7% of the total variance in the training set (*R* ^2^ = 0.927), and there was a strong correlation between predicted age and chronological age (Spearman’s rho = 0.963) with an MAE from chronological age of 3.682 years and an RMSE of 4.783 years (Figure 4). In the testing set, the RFR model also showed a strong correlation between predicted and chronological ages (Spearman’s rho = 0.77) with an MAE of 6.84 years. Additionally, we also constructed models based on a single type of biomarkers, miRNAs or circRNAs only. Results showed that the combination of these two types of biomarkers obtained significantly higher accuracy than using only one type of them, which achieved an MAE of 8.1–10.9 years in the testing set (circRNA only) and of 9.1–12.6 years (miRNA only).

**TABLE 1:** Comparison of different prediction models.

Models	Training set			Testing set		
	MAE (years)	RMSE (years)	rho	MAE (years)	RMSE (years)	rho
Tree	6.348	8.476	0.828	7.985	10.217	0.652
Bagging	6.384	8.047	0.859	7.455	9.002	0.715
RFR	3.680	4.878	0.981	6.840	8.422	0.765
SVR	6.536	9.106	0.805	7.281	8.999	0.717
XGBoost	4.260	5.916	0.937	7.675	10.391	0.753

**FIGURE 5:**
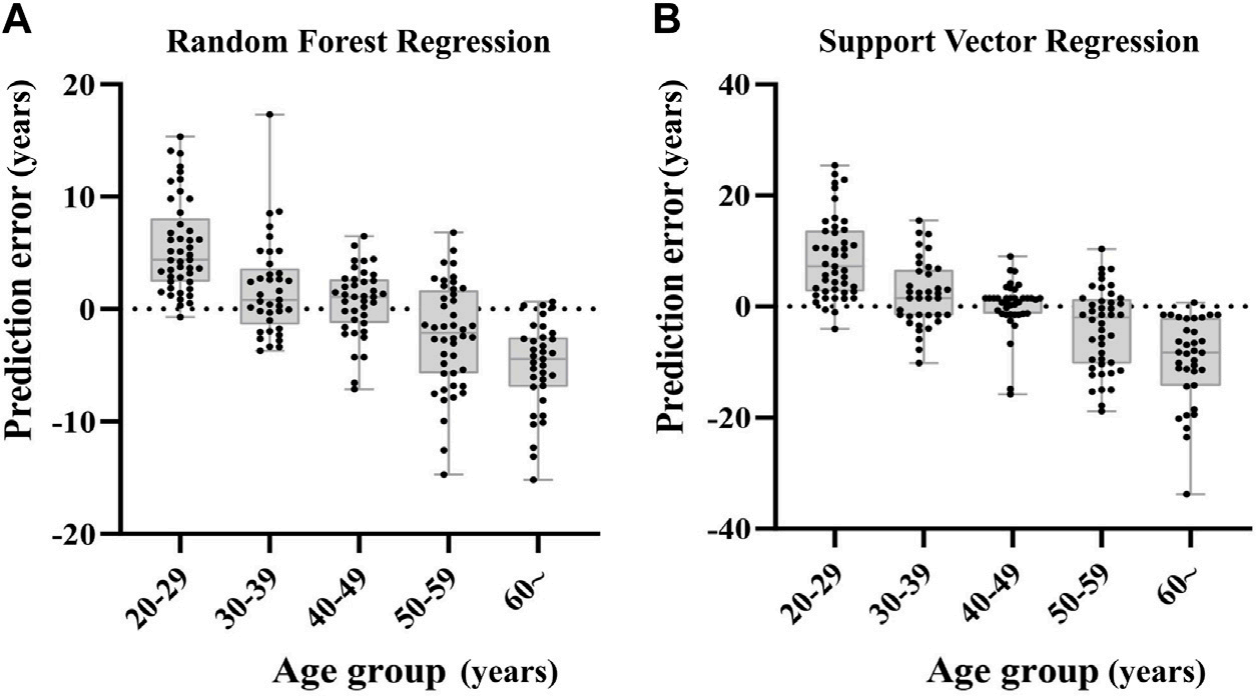
Deviations of the prediction from the chronological age in RFR model and SVR model. The subjects of the younger age group are rather overestimated in comparison to older individuals that tend to be underestimated.

We grouped the samples into five age categories (20s, 30s, 40s, 50s, 60s or more), taking RFR model and SVR model for example. The prediction errors in older individuals were bigger, and the predicted age of older samples was rather underestimated, whereas the age prediction of young individuals was overestimated (Figure 5).

As females have longer lifespans than males, we investigated if a sex-dependent difference in the selected ncRNAs expression exists. However, no statistically significant difference was observed between ncRNA expression levels and sexes (Mann Whitney test, *p* > 0.05). This result indicated that ncRNA expression in our study is stronger associated with age as compared to sex. In addition, we also performed modeling using female samples and male samples separately. The age prediction of male individuals was slightly superior to female individuals with MAEs of 6.8–8.5 years for males and of 6.4–11.2 years for females in the testing set, respectively (Table 2). Sex had a slight influence on the accuracy of the models in the current study.

**TABLE 2:** Age prediction performance of different models between female and male individuals (the bold values represent a lower MAE value).

Models	Female (n = 102)				Male (n = 98)			
	Training set		Testing set		Training set		Testing set	
	rho	MAE (years)	rho	MAE (years)	rho	MAE (years)	rho	MAE (years)
Tree	0.752	8.191	0.84	6.410	0.813	5.799	0.506	7.596
Bagging	0.828	7.581	0.787	7.969	0.855	5.837	0.569	6.796
RFR	0.962	4.403	0.864	6.486	0.965	3.421	0.447	7.736
SVR	0.812	7.074	0.887	7.440	0.807	6.071	0.480	8.464
XGBoost	0.959	3.850	0.595	11.247	0.992	1.575	0.612	8.283

## 4 Discussion

MiRNAs and circRNAs are highly abundant, stable, and have been demonstrated to change with increasing age and development, sparking interest in their use as potential biomarkers to predict chronological age. In the current study, we explored how the global expression of miRNAs and circRNAs change in normal human aging in peripheral blood samples collected from healthy individuals. We identified age-associated ncRNAs (11 miRNAs and four circRNAs) through NGS and integrated bioinformatics methods. Age prediction models were developed based on these 15 ncRNAs using machine learning methods including regression tree, bagging, RFR, SVR and XGBoost. Compared to previous miRNA-based age prediction models, which reported MAE for their models ranging from 6.56 to 9.262 years using 6 miRNAs (Fang et al., 2020), and our previous study of circRNA-based prediction models with MAE 8.77–12.19 years using 5 circRNAs (Wang et al., 2022), models in this study exhibit higher accuracy in age prediction with MAE of 6.8–7.9 years by combining age-related miRNAs and circRNAs biomarkers.

It had been shown previously that some of the age-related miRNAs used in this study upregulate with increased age in different organisms, tissues, or cell types. For instance, the aged Ames mice study showed increased expression of miR-27a (Bates et al., 2010). The expression level of miR-222 was significantly altered during aging in the human population reported by several authors. A study on serum samples from healthy subjects of different age groups identified upregulation of miR-222 in an age-dependent manner (Zhang et al., 2015). Hanna and others found that miR-27a and miR-222 were consistently upregulated in replicative senescent cells. Additionally, miR-339-5p, miR-222, miR-27a, miR-330 and miR-107 were associated with age-related diseases such as Alzheimer’s Disease (AD), Parkinson’s Disease (PD) and Mild cognitive impairment (MCI) (Kumar et al., 2017; Reddy et al., 2017; John et al., 2020). MiR-199a-5p and miR-27a were found up-regulated expressed in the photoaging process and miR-330 was closely associated with brain development (Choi et al., 2016). Some miRNAs were reported to have biological relevance with aging but in a different manner. For example, miR-107 was down-regulated from infancy to childhood and had a diminishing expression with age from young (≤35 years old) to old adults (≥35 years old) (Lai et al., 2014). Pathway enrichment analysis of age-related miRNAs’ target genes in this work showed they are significantly enriched in the cell senescence pathway and FoxO signaling pathway which is involved in many cellular physiological events such as longevity (Martins et al., 2016; Green et al., 2022). This finding contributed to our understanding of the pathways of aging, however, further experiments are required to confirm the underlying mechanisms of aging.

Three datasets analyzed in this study were derived from different studies. Dataset GSE89042 was conducted by Maider and others (Munoz-Culla et al., 2017), they analyzed the expression of small non-coding RNA (sncRNA, microRNA and small nucleolar RNA -snoRNA-) by microarrays in leucocytes from healthy donors of different ages, ranging from 24 to 79 years old. They performed a sliding window analysis to select age-related sncRNAs and then observed that the expression of 69 sncRNAs changed progressively with chronological age. In this study, we filtered probes to exclusively remain miRNAs, which we are interested in. Most age-related miRNAs screened in this study were consistent with the study conducted by Maider et al. But miR-1281 which showed age-associated expression in RT-qPCR in our study did not show any difference in its expression between young and old groups in the validation set of Maider’s research. Dataset E-MTAB-3303 contained 211 RNA samples, including 128 schizophrenia subjects and 83 healthy controls (Wang et al., 2015). We extracted data from controls for age-related analyses. Dataset E-MTAB-1231 included three age groups of individuals, young (<40 years old), octogenarians and centenarians (Serna et al., 2012). We regrouped them into young vs. old to explore the general patterns of molecular change between young and old individuals. However, Serna et al. found that centenarians upregulate the expression of sncRNAs whereas octogenarians downregulate it when compared to young people. They supposed that striking maintenance of homeostatic mechanisms and healthy aging of a population with extreme longevity may be explained by their specific characteristic of gene expression. This conclusion provides the basis for considering the age prediction of centenarians separately in the future.

CircRNA molecules offer a promising field in aging research, however, its forensic applications are still in the preliminary stage. In our work, we uncovered a trend for increased circRNA expression in the blood of old individuals *versus* young. We classified the circRNAs into three classes: age-limited expression-young only, age-limited expression-old only, and age-related expression. The top five differentially expressed circRNAs in the classes with age-limited or age-related expression were validated using qPCR. Four circRNAs confirmed in a validation set were derived from different host genes. Although there is no research about hsa_circ_0104147, its host gene, *HERC1*, encodes a member of the *HERC* protein family which may be involved in membrane transport processes. Larsson et al. reported that variants in *HERC1* may influence normal neuronal pattern development (Larsson et al., 2011). Hsa_circ_0005400 originated from the gene ACAP2. Research showed knockdown of ACAP2 blocks apoptosis in cancer cells in response to the chemotherapeutic antimetabolite 5-fluorouracil and ACAP2 expression was down-regulated in some esophageal cancers, leukemias and lymphomas (Sullivan et al., 2015). Several studies demonstrated that circACAP2 facilitates the proliferation of cancers such as colorectal cancer and breast cancer by targeting multiple miRNAs (Zhao et al., 2020; Zhang et al., 2021). The host gene of CircMYH11 is *MYH11*, which was confirmed that non-coding variants in *MYH11* are associated with dementia in women. Multiethnic studies identified ten genes including *MYH11* are differentially expressed in the context of Alzheimer’s disease (AD) (Blue et al., 2021). The product of the gene PPP2R5A which is the parental gene of circPPP2R5A is implicated in the negative control of cell growth and division. PPP2R5A is also reported to be involved in regulating DNA repair and apoptosis through many pathways (Mao et al., 2018). It is well known that cancer, dementia and AD are the most important age-related disorder and occur in elderly individuals. Intriguingly, age-related circRNA screened in our study, their parental genes play a role in multiple cell activities and age-related disorders. Age-related genes may regulate the aging process by mediating numerous ncRNAs. However, the regulation network of aging is still not fully elucidated.

The role of ncRNAs in predicting chronological age for forensic applications is still a relatively new area of research. Since various biological age predictors reflect different aspects of the aging process, considering multiple combinations of different types of predictors may shed light on the aging process and provide a further understanding of healthy aging (Jylhava et al., 2017). Previously published studies combined sjTRECs, mRNA and DNA methylation. sjTRECs-based methods alone give MAE of about 10 years, while DNA methylation-based methods give MAE of about 3–5 years. Even higher predictive accuracy with an MAE of about 3.3 years was exhibited when combining sjTRECs and DNA methylation (Cho et al., 2017), while an MAE of 4.6 years was shown by a combination of DNA methylation and mRNA predictors (Zubakov et al., 2016). In the present work, we first simultaneously examined miRNAs and circRNAs predictors and constructed age prediction models using these two types of biomarkers. Results showed that the combination of these two types of biomarkers obtained significantly higher accuracy with MAE of about 6–7 years than using only one type of them, which achieved MAE of 8.1–10.9 years in the testing set (circRNA only) and of 9.1–12.6 years (miRNA only). Age group analyses exhibit a tendency to overestimate age in young adults and to underestimate it in older ones. This trend has already been reported by several previous studies (Beheshti et al., 2019) (de Lange and Cole, 2020) (Liang et al., 2019). It can be explained as a result of the statistics used for the development of this class of predictive age equations. AG de Lange et al. explained this phenomenon as the limiting case where a model is unable to predict age based on the input features (de Lange et al., 2022).

Models in our study are currently restricted to the sample size of 200 samples and an age range of 20–80 years old that did not incorporate children and adolescents. ncRNAs are thought to modulate the expression of a variety of human genetic codes involving not only the aging process but also the developmental process. Thus, more under-aged-specific, adult-specific and elderly-specific predictors are needed to investigate in future work. Broadening the predictable age range may exhibit the regulation mechanisms of biological molecules in different age stages. Although studies reported that sex hormones can modulate ncRNA synthesis and/or secretion (Perez-Cremades et al., 2018; Sheinerman et al., 2018), sex had no statistically significant difference in the expression levels of ncRNAs and had a small effect on age prediction in our study. More samples per sex may facilitate exploring the rule of expression patterns of ncRNAs between males and females. Compared to DNA methylation markers, the accuracy of age estimation based on ncRNA markers are still needed to improve. Additionally, the use of predictors for other populations from different ethnic backgrounds is needed to be explored. The correlations between age and circRNA expression levels were significantly higher than that of miRNAs. We assume that miRNAs selected from online datasets conducted using other populations are perhaps not suitable biomarkers for the Chinese population. Further profound analyses are still needed to assess the application of the current estimation models for other specimens like saliva or semen and the application of low template RNA samples.

## Data Availability

The datasets presented in this study can be found in online repositories. The names of the repository/repositories and accession number(s) can be found in the article/Supplementary Material.
