# Supplementary material for: Forensic age estimation from human blood using age-related microRNAs and circular RNAs markers
Source: Front Genet. 2022 Nov 22;13:1031806. doi: 10.3389/fgene.2022.1031806 (PMC9732945; doi:10.3389/fgene.2022.1031806)
Supplement: Supplementary file 1 [file DataSheet1.pdf]

Supplementary Material

1 Supplementary Figures and Tables

1.1 Supplementary Figures

Supplementary Figure 1. The age distribution and sex distribution of 248 samples.

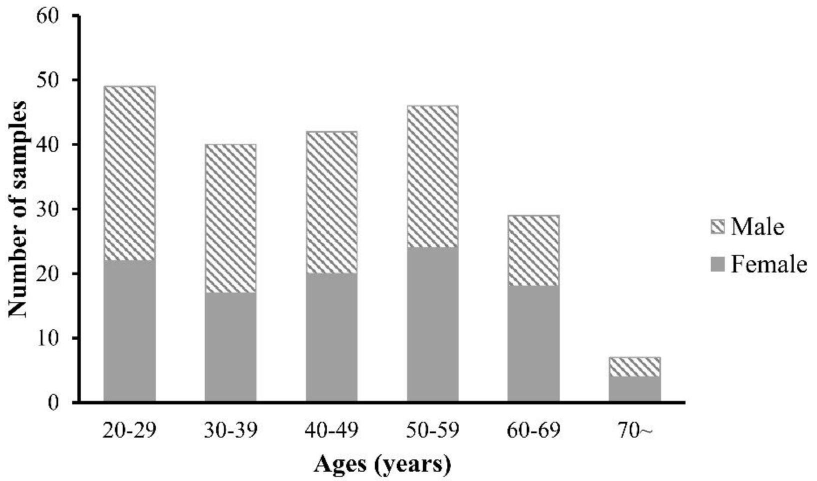

Supplementary Figure 2. Normalization of gene expression. (A-B) Normalization of the E-MTAB-1231 dataset. **(C-D)** Normalization of the E-MTAB-3303 dataset. Boxplots on the left represent data before normalization, and ones on the right represent data after normalization.

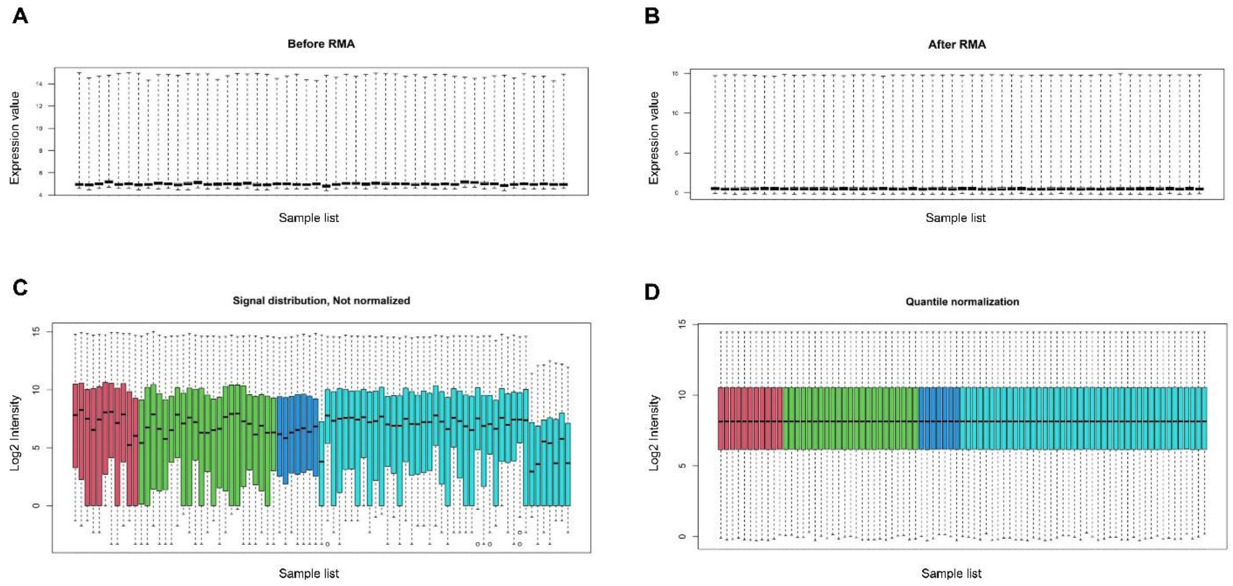

1.2 Supplementary Tables

Supplementary Table 1. Details of the ArrayExpress and GEO datasets.

EBI/GEO	Tissue (N)	Age-related miRNAs	Up	Down	Age	Reference
**E-MTAB-3303**	PBMC (83)	55	40	15	17-69	Xi Wang et al. (2015)
**E-MTAB-1231**	Mononuclear (50)	117	117	0	24-104	Serna E et al. (2013)
**GSE89042**	Leucocytes (38)	40	30	10	24-79	Muñoz-Culla M et al. (2017)

Supplementary Table 2. Primers used in our study.

Targets	Primers (5' to 3')
U6	Provided by TaKaRa Bio Inc
hsa-miR-107	F: CAGCAGCATTGTACAGGGCTATCA
hsa-miR-339-5p	F: TCCCTGTCCTCCAGGAGCT
hsa-miR-940	F: TATATAAGGCAGGGCCCCCG
hsa-miR-423-3p	F: TAGCTCGGTCTGAGGCCC
hsa-miR-27a-3p	F: CCTTCACAGTGGCTAAGTTCCGC
hsa-miR-652-3p	F: TAATGGCGCCACTAGGGTTGTG
hsa-miR-199a-5p	F: GCCCAGTGTTCAGACTACCTGTTC
hsa-miR-222-3p	F: CCAGCTACATCTGGCTACTGGG
hsa-miR-1281	F: TTCGCCTCCTCCTCTCCC
hsa-miR-425-3p	Designed and synthesized by RiboBio
hsa-miR-1228-3p	F: TATATATCACACCTGCCTCGCCCC
hsa-miR-378a-3p	F: ACTGGACTTGGAGTCAGAAGGC
hsa-miR-330-3p	Designed and synthesized by RiboBio
hsa-miR-345-5p	F: GCTGACTCCTAGTCCAGGGC
hsa-miR-93-3p	F: ACTGCTGAGCTAGCACTTCCC

hsa-miR-491-5p	F: ATAGTGGGGAACCCTTCCATGAGG
hsa-miR-500a-3p	F: TATGCACCTGGGCAAGGATTCTG
hsa-miR-744-5p	F: TGCGGGGCTAGGGCTAA
18S rRNA	F: CTTGCTGGTCTGATTGTCGTT<br>R: CTCGTCTGTTAGGTGGATGCT
hsa_circ_0007099	F: CTGCTGTGCTGTACGTGATC<br>R: AGAGTTGTACAGTTGGGCCG
hsa_circ_0104147	F: GTGGTTCATGGGCTTTTGGA<br>R: TTAGGTCCAGCAGCACAGAA
hsa_circ_0005400	F: CCACCAACATTCTGACAGCA<br>R: CAAAGTTCTGAAGCTGTGCCT
hsa_circ_0130015	F: GGAACCAGAAAAGATACGAGGC<br>R: TCAGTGCGGTAGTCTTTTCTT
CircMYH11	F: TGCCTCCCAGATTCAAGTGA<br>R: AAGAGTGACATCAGCAGCCT
CircNFATC3	F: AATATGTCAGCCAGCTCCTG<br>R: CTGGTAAAATGCATGAGGTCGT
CircPPP2R5A	F: CCCAGAGAACGTGACTTCCT<br>R: TCGTTTTGCAATGCTAGGCT
CircCAMLG	Failed in primer design
CircNCOA5	Failed in primer design
Novel_circ_0113405	F: CAATGTGCCAGCTCCATCTT<br>R: TTCCTTGTCCTGGGCATTCT

CircCNTRL	Failed in primer design
CircBMPR2	Failed in primer design

Supplementary Table 3. Expression patterns and locations on human chromosomes of age-related miRNAs.

**miRNA**	**Expression**	**Location on human chromosome**
hsa-mir-107	Up	chr10: 89592747-89592827 [-]
hsa-mir-339	Up	chr7: 1022933-1023026 [-]
hsa-mir-146a	Controversial	chr5: 160485352-160485450 [+]
hsa-mir-940	Up	chr16: 2271747-2271840 [+]
hsa-mir-423-5p	Controversial	chr17: 30117079-30117172 [+]
hsa-mir-423-3p	Up	chr17: 30117079-30117172 [+]
hsa-mir-27a	Up	chr19: 13836440-13836517 [-]
hsa-mir-652	Up	chrX: 110055329-110055426 [+]
hsa-mir-199a	Up	chr19: 10817426-10817496 [-]
hsa-mir-222	Up	chrX: 45747015-45747124 [-]
hsa-let-7a	Controversial	chr9: 94175957-94176036 [+]
hsa-mir-25	Controversial	chr7: 100093560-100093643 [-]
hsa-let-7i	Controversial	chr12: 62603686-62603769 [+]
hsa-let-7g	Controversial	chr3: 52268278-52268361 [-]
hsa-let-7c	Controversial	chr21: 16539828-16539911 [+]
hsa-mir-1281	Up	chr22: 41092513-41092566 [+]

hsa-mir-425	Up	chr3: 49020148-49020234 [-]
hsa-mir-1228	Up	chr12: 57194504-57194576 [+]
hsa-mir-378	Up	chr5: 149732825-149732890 [+]
hsa-mir-944	Controversial	chr3: 189829922-189830009 [+]
hsa-mir-330	Up	chr19: 45638994-45639087 [-]
hsa-mir-345	Up	chr14: 100307859-100307956 [+]
hsa-mir-93	Up	chr7: 100093768-100093847 [-]
hsa-mir-491	Up	chr9: 20716105-20716188 [+]
hsa-mir-500a	Up	chrX: 50008431-50008514 [+]
hsa-mir-744	Up	chr17: 12081899-12081996 [+]

Supplementary Table 4. GO analysis of age-related miRNAs' target genes (show top 10 terms).

Terms	Description	Count	*P*-adj Value
**GO:0048872**	homeostasis of number of cells	19	0.003081005
**GO:0034101**	erythrocyte homeostasis	13	0.003081005
**GO:0002262**	myeloid cell homeostasis	14	0.003182789
**GO:0030218**	erythrocyte differentiation	12	0.004388056
**GO:0030099**	myeloid cell differentiation	24	0.00592676
**GO:0071214**	cellular response to abiotic stimulus	20	0.012491776
**GO:0104004**	cellular response to environmental stimulus	20	0.012491776
**GO:0032648**	regulation of interferon-beta production	7	0.026544388
**GO:0009314**	response to radiation	23	0.028027942
**GO:0032608**	interferon-beta production	7	0.028027942

Supplementary Table 5. KEGG pathway analysis of age-related miRNAs' target genes (show top 10).

**Pathway**	**ID**	**Count**	***P*-adj Value**
**Cellular senescence**	hsa04218	15	0.001624013
**Non-small cell lung cancer**	hsa05223	8	0.021617616
**FOXO signaling pathway**	hsa04068	11	0.021617616
**Viral carcinogenesis**	hsa05203	14	0.021617616
**Pancreatic cancer**	hsa05212	8	0.021617616
**Proteoglycans in cancer**	hsa05205	14	0.021617616
**Sphingolipid signaling pathway**	hsa04071	10	0.023282768
**Cell cycle**	hsa04110	10	0.024770907
**Breast cancer**	hsa05224	11	0.024770907
**Human T-cell leukemia virus 1 infection**	hsa05166	14	0.024770907
